# Control of methicillin-resistant *Staphylococcus aureus* (MRSA): the impact of active surveillance for MRSA in a non-acute hospital in Madrid (Spain)

**DOI:** 10.1186/1753-6561-5-S6-P8

**Published:** 2011-06-29

**Authors:** C Bischofberger, L Garcia-Picazo, C Garcia-Gonzalez, M Alonso, Á Asensio

**Affiliations:** 1 Medicina Preventiva, Hospital de El Escorial / Guadarrama, Guadarrama, Madrid, Spain; 2 Microbiología, Hospital El Escorial, Madrid, Spain; 3 Medicina Interna, Hospital de Guadarrama, Madrid, Spain; 4 Medicina Preventiva, Hospital Universitario Puerta de Hierro, Madrid, Spain

## Introduction / objectives

Because of the frequent interchange of patients between acute hospitals and rehabilitation hospitals, infections caused by multidrug-resistant bacteria will continue to emerge. There are limited data concerning the epidemiology of nosocomial infection among patients admitted for rehabilitation in non acute hospitals after stabilization of an acute surgical or neurological event. Recognition of such threats has prompted new interest in the prevention and control of infections associated with those health-care facilities.

## Objective

To evaluate the impact of an intervention on rates of methicillin-resistant Staphylococcus aureus (MRSA) colonization or infection in a geriatric-rehabilitation hospital.

## Methods

### Design

Quasi-experimental analysis before/after.

### Setting

A 160 bed geriatric-rehabilitation hospital in Guadarrama (Madrid), Spain.

### Patients

All patients admitted to the hospital during two periods: 2003-2004 and 2010.

## Methods

A multimodal intervention consisting on active surveillance for MRSA in all patients admitted and a comprehensive hand hygiene promotion programme is studied.

## Results

The preintervention rate of MRSA colonization or infection was 1.02 cases per 1,000 patient-days (95% confidence interval {CI}, 0.93-1.25 cases per 1,000 patient-days). The rate decreased significantly to 0.21 cases per 1,000 patient-days (95% CI, 0.12-0.45 cases per 1,000 patient-days) after the intervention.

## Disclosure of interest

None declared.

